# Microglia in Alzheimer’s Disease: A Target for Therapeutic Intervention

**DOI:** 10.3389/fncel.2021.749587

**Published:** 2021-11-24

**Authors:** Guimei Zhang, Zicheng Wang, Huiling Hu, Meng Zhao, Li Sun

**Affiliations:** ^1^Department of Neurology and Neuroscience Center, The First Hospital of Jilin University, Jilin University, Changchun, China; ^2^Department of Intensive Care Unit, The Affiliated Hospital of Qingdao University, Shandong, China

**Keywords:** Alzheimer’s disease, β-amyloid, microglia, neuroinflammation, tau

## Abstract

Alzheimer’s disease (AD) is one of the most common types of age-related dementia worldwide. In addition to extracellular amyloid plaques and intracellular neurofibrillary tangles, dysregulated microglia also play deleterious roles in the AD pathogenesis. Numerous studies have demonstrated that unbridled microglial activity induces a chronic neuroinflammatory environment, promotes β-amyloid accumulation and tau pathology, and impairs microglia-associated mitophagy. Thus, targeting microglia may pave the way for new therapeutic interventions. This review provides a thorough overview of the pathophysiological role of the microglia in AD and illustrates the potential avenues for microglia-targeted therapies, including microglial modification, immunoreceptors, and anti-inflammatory drugs.

## Introduction

### Alzheimer’s Disease

Alzheimer’s disease (AD) is one of the most common types of age-related dementia worldwide. It includes progressive cognitive deficits, impaired daily activities, and abnormal behavioral and psychological symptoms (Long and Holtzman, 2019). Due to the largely unknown etiology of AD and lack of efficient therapeutic approaches, a tremendous pressure has forced researchers to delve further into molecular and cellular pathways in an attempt to elucidate the pathogenesis of AD. In addition to extracellular amyloid plaques and intracellular neurofibrillary tangles (NFTs), gliosis and neuroinflammation also play deleterious roles in AD, implicating microglia in the disease process. Microglia are the main innate immune cells in the central nervous system (CNS) and act as guardians by responding to CNS infection or damage. Numerous studies have demonstrated that microglia have dual functions in the pathological process of AD and act in a context-dependent manner. The moderate activation of microglia under β-amyloid (Aβ) stimulation has neuroprotective effects. Some pattern recognition receptors expressed on the surface of microglia interact with neurotoxic Aβ to promote Aβ clearance from the brain. However, unbridled microglial activity can create a chronic neuroinflammatory environment, exacerbating neuronal and synaptic loss, tau pathology, and cognitive decline. Accumulating evidence suggests that dysfunctional microglia may actively contribute to AD pathogenesis. Thus, examining context-dependent microglial reactions may reveal potential avenues for the treatment of AD.

## Development and Function of Microglia

Since the mystery of microglia was unveiled one century ago (Sierra et al., 2016), a longstanding debate on the origin of microglia began. Owing to various periods of brain development (embryonic, postnatal, and adult microglia) and ever-changing surroundings, microglial ontogeny has not yet been established. Contrary to astrocytes and oligodendrocytes derived from the neuroectoderm, microglia are tissue-resident macrophages that originate from the mesoderm during embryonic development (Sierra et al., 2016). The most consensual hypothesis is that microglia are derived from progenitors, that originate from the yolk sac (YS; Cuadros et al., 1993; Alliot et al., 1999; Bertrand et al., 2005; Gomez Perdiguero et al., 2015; Askew et al., 2017). Cuadros et al. (1993) suggested that ameboid microglia are derived from the YS extraembryonic precursors in the early stages of the avian CNS, independent of CNS vascularization. This result was later confirmed by Alliot et al. (1999) who reported potential microglial progenitors in the YS on murine embryonic day (E) 8. The extraembryonic YS is the first source of hematopoietic progenitors before blood circulation is established (E8.5; Palis et al., 1999). These hematopoietic progenitors then expand into the fetal liver, rapidly initiating intraembryonic hematopoiesis. Primitive and definitive hematopoietic progenitors arise from the YS between E7.0 and E9.5, migrate through the bloodstream, and expand in the fetal liver after E9.5 (Lux et al., 2008).

To date, the contribution of YS-derived cells to macrophage pools or microglia has been intensely investigated and is gaining increasing attention. Tissue macrophages are thought to originate from hematopoietic stem cells. However, Schulz et al. (2012) found a possible YS origin of macrophages. They reported a discrepancy in the genotype between a lineage of YS-derived tissue macrophages and the progeny of hematopoietic stem cells. They observed that the YS-derived cell population, including several tissue macrophages (microglia) can proliferate and renew them independently from the transcription factor Myb, which is required for hematopoietic stem cells development. Furthermore, Bertrand et al. (2005) found three pathways of macrophage maturation during the development of YS hematopoiesis. The first wave of macrophage population detected in the YS comprised maternal-derived cells with a mature phenotype of CD45^+^Mac-1^+^F4/80^+^. These macrophages observed in the early YS at 7.5–8 postcoital days with transient contribution to mature macrophages. The second wave population comprising monopotent-derived precursors appear later, at postcoital day 8 (2- to 4-somite stage), exhibiting a c-Kit^+^CD45^−^ phenotype. Finally, erythromyeloid precursors constituted the third wave immediately following the second wave at the 4- to 6-somite stage with a similar phenotype (CD45^−^c-Kit^+^). These YS-derived macrophages may be instrumental in establishing resident tissue macrophages, including microglia. Recently, advances in fate mapping analysis have also provided further convincing evidence about the YS origin of microglia (Ginhoux et al., 2010; Gomez Perdiguero et al., 2015). Adequate understanding of microglial origin will help us achieve the therapeutic goal of repairing dysfunctional microglia.

**Figure 1:**
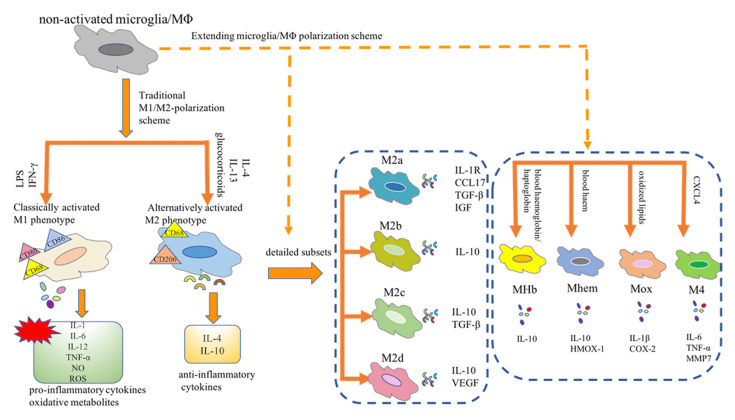
Microglia/Macrophage polarization subtypes. Traditionally, the M1 or M2 phenotype is considered as the extreme activation state of macrophages/microglia in response to different microenvironmental stimuli. Notably, novel observations have revealed the detailed macrophage subsets: Mhem, Mox, MHb, and M4. Moreover, the M2 macrophages have been further subdivided into the M2a, M2b, M2c, and M2d subtypes. MΦ, macrophages; LPS, lipopolysaccharide; IFN-γ, interferon-γ; IL, interleukin; TNF-α, tumor necrosis factor-α; NO, nitric oxide; ROS, reactive oxygen species; CCL17, CC chemokine ligand 17; IGF, insulin-like growth factor; TGF-β, transforming growth factor-β; VEGF, vascular endothelial growth factor; HMOX-1, heme oxygenase-1; COX2, cyclooxygenase-2; CXCL-4, chemokine (C-X-C motif) ligand (CXCL) 4; MMP7, matrix metalloproteinase 7.

As innate immune sentinels, microglia are important players in CNS homeostasis. In the *in vitro* settings, distinct phenotypic subpopulations of microglia are considered to independently perform their biological functions in response to different environmental cues (Martinez et al., 2008). For example, in response to stimulation with interferon (IFN)-γ and lipopolysaccharide (LPS), microglia shift from a quiescent to an activated state, which is referred to as classically activated M1 microglia. These M1 microglia release a plethora of pro-inflammatory mediators, contributing to the vicious cycle of chronic inflammation. Conversely, alternatively activated M2 phenotypes, stimulated by interleukin (IL)-4, play a neuroprotective role by releasing trophic factors, resolving inflammation, and phagocytosing harmful substances. Overall, in the complex pathological settings, activated microglia undergo either pro-inflammatory (M1) or anti-inflammatory (M2) phenotypic alterations; thus, these cells play a dualistic role in the battle of disease progression. Microglia are tissue-resident macrophages and studies of microglial polarization schemes often focus on macrophages. Traditionally, the M1 or M2 phenotype is considered as the extreme activation state of macrophages/microglia in response to different microenvironmental stimuli. However, with the advent of novel technologies, the M1/M2 polarization model is now considered less valid (Martinez et al., 2008; Xue et al., 2014; Ransohoff, 2016). A transcriptome-based network analysis extended the current M1/M2-polarization scheme by revealing a spectrum model with at least nine macrophage activation patterns (Xue et al., 2014). Notably, novel observations have revealed the detailed macrophage subsets: Mhem, Mox, MHb, and M4 (Chinetti-Gbaguidi et al., 2015; Nakai, 2021). Moreover, the M2 macrophages have been further subdivided into the M2a, M2b, M2c, and M2d subtypes (Chinetti-Gbaguidi et al., 2015; Nakai, 2021). These diverse macrophage subtypes respond to different microenvironmental stimuli and surface markers and thus have distinct biological functions (Figure 1). Most studies aimed at describing the phenotypes of macrophages/microglia are conducted mainly *in vitro*. However, whether all these subsets can be found *in vivo* remains uncertain. Understanding the function of these macrophages/microglia subtypes and their contribution to health or disease contributes to the design of novel strategies to hinder or reverse disease progression.

**Figure 2:**
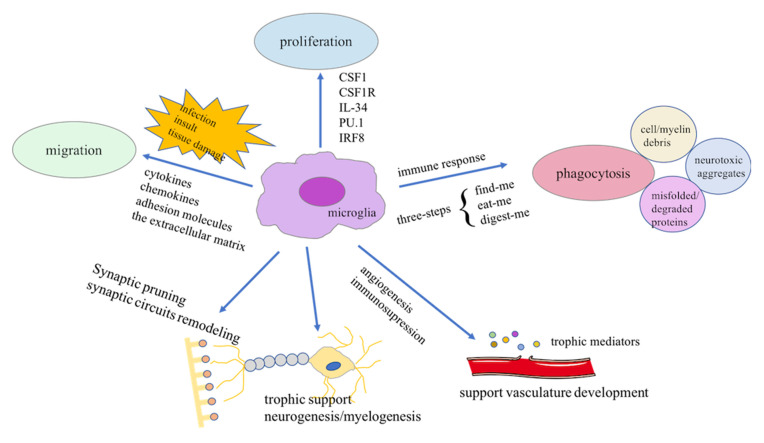
Physiological functions of microglia. Scheme illustrating the diverse functions of microglia during development and microenvironmental stimuli. CSF1, colony stimulating factor 1; CSF1R, CSF1 receptor; IRF8, interferon regulatory factor 8.

Since the active role of microglia as macrophages is well established, the main functional features of microglia, including proliferation, migration, and phagocytosis, have also been characterized (Sierra et al., 2016). Microglia undergo a wave of proliferation to maintain their stable density after arising from the YS. Recent evidence indicates that adult microglia can also maintain a stable number of cells according to their own population dynamics, rather than by peripherally infiltrating monocytes (Askew et al., 2017). Microglial homeostasis can be achieved by spatiotemporally coupled proliferation and apoptosis during their lifetime (Lawson et al., 1992; Askew et al., 2017). Direct evidence has identified some molecular regulators of microglial proliferation, including colony stimulating factor 1 (CSF1), CSF1 receptor (CSF1R), IL-34, the transcription factor PU.1, and IFN regulatory factor 8 (IRF8; Mizuno et al., 2011; Minten et al., 2012; Gómez-Nicola et al., 2013; Askew et al., 2017). To maintain CNS homeostasis, microglia vigilantly monitor the microenvironment with their extremely rapid mobility and highly dynamic motility. Based on well-coordinated cellular and molecular signaling (Franco-Bocanegra et al., 2019; Smolders et al., 2019), microglia rapidly exhibit “chemotactic motility” when encountering infection, insult, or tissue damage. After sensing potential hazards, microglia move in the direction of the cue, under the control of a series of events. During mobility, microglial migration speed can be regulated by a series of molecular signaling pathways, such as cytokines, chemokines, adhesion molecules, and the extracellular matrix (Milner and Campbell, 2002, 2003; Carbonell et al., 2005; Arnò et al., 2014). Microglia change their shape to adapt to their environment, which relies on the versatility of cytoskeletal proteins, the cross-linked protein system, and the sophisticated system of chemotactic membrane receptors (Franco-Bocanegra et al., 2019). The most studied characteristic of microglia is phagocytosis, which relies on a three-step model: the initial “find-me” step, the subsequent “eat-me” step, and the final “digest-me” step (Sierra et al., 2013). Under physiological conditions, some chemoattractant molecules released by damaged or dead cells serve as “find-me” signals to initiate the chemotactic motility of microglia. Under the coordination of some receptors expressed on the surface of microglia and their ligands, an engulfment synapse is formed, which subsequently internalizes the potentially harmful particles (Ravichandran, 2010). These harmful particles are eventually degraded in phagolysosomes, which are formed by the fusion of phagosomes with lysosomes and other organelles. In addition to their proliferation, migration, and phagocytotic activity, microglia also participate in synaptic plasticity (Paolicelli et al., 2011; Kettenmann et al., 2013; Schafer et al., 2013), developmental sculpting of neural circuits (Schafer et al., 2012; Frost and Schafer, 2016), immune response (Shaked et al., 2004), and vasculature development (Dudvarski Stankovic et al., 2016; Figure 2). Taken together, the functional diversity of microglia plays a critical role in maintaining CNS homeostasis.

## Microglia and AD

**Figure 3:**
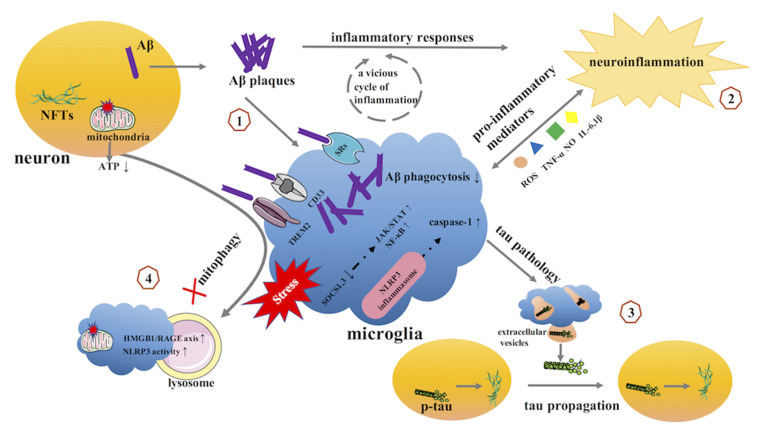
The potential role of microglia in AD. (1) microglia and Aβ. (2) microglia and neuroinflammation. (3) microglia and tau pathology. (4) microglia and mitophagy. AD, Alzheimer’s disease; Aβ, β-amyloid; NFTs, neurofibrillary tangles; SRs, scavenger receptors; TREM2, triggering receptor expressed on myeloid cells 2; SOCS, suppressor of cytokine signaling; JAK, Janus kinase; STAT, signal transducer and activator of transcription; NF-κB, Nuclear Factor-κB; NLRP3, NOD-like receptor family pyrin domain containing 3.

Microglia play important roles in the homeostatic brain, including surveying the entire brain parenchyma, protecting the CNS from pathogenic attacks, and preserving CNS integrity. However, the homeostatic functions of these cells are lost in AD. Accumulating evidence implicates diseased or weakened microglia as important mediators in AD pathology (Table 1, Figure 3).

**Table 1:** The pathological and protective mechanisms of microglia involvement in AD.

Pathological events	Molecules/Genes	Impact on microglial function	References
Microglia and Aβ	SR-A	promote microglia adhesion to Aβ and increase microglia uptake of Aβ	Frenkel et al. (2013)
	CD36	play a dichotomous role in Aβ phagocytosis	Kim et al. (2017)	
	RAGE	play a dichotomous role in Aβ phagocytosis	Deane et al. (2012)
	*APOE*	*APOE ε4* genotype is related to a dampened microglial response to amyloid plaques	Nguyen et al. (2020)
	*CR1*	promote the phagocytosis of Aβ by microglia	Crehan et al. (2013)
	*TREM2*	play a dichotomous role in Aβ phagocytosis	Ulland et al. (2017)
	*CD33*	reduce the phagocytosis of Aβ by microglia	Yin et al. (2017)
	*ABCA7*	promote the phagocytosis of Aβ by microglia	Aikawa et al. (2019)
Microglia and Neuroinflammation	NLRP3	aggravate microglia-mediated inflammatory response	Heneka et al. (2013)
	SOCS	play a protective role by balancing the inflammatory response	Ruganzu et al. (2021)
	CX3CR1	*CX3CR*1 deficiency aggravates tau phosphorylation	Cho et al. (2011)
Microglia and Tau Pathology	CSF1R	inhibition of CSF1R leads to an attenuation of tau-induced neurodegeneration	Mancuso et al. (2019a); Mancuso et al. (2019b)
	*APOE*	*APOE ε4* markedly exacerbates tau-mediated neurodegeneration	Shi et al. (2017)
	*TREM2*	loss of TREM2 function promotes seeding and spreading of neuronal plaque tau aggregates.	Leyns et al. (2019)
Microglia and Mitophagy	HMGB1/RAGE signaling pathways	play an important role in blocking late-stage mitophagy in microglia	Zhang et al. (2020)

*SR, scavenger receptors; RAGE, the receptor for advanced glycation end products; APOE, Apolipoprotein E; CR1, complement receptor 1; TREM2, triggering receptor expressed on myeloid cells 2; ABCA7, ATP-binding cassette transporter A7; NLRP3, the NOD-like receptor family pyrin domain-containing 3; SOCS, suppressors of cytokine signaling; CX3CR1, CX3C chemokine receptor 1; CSF1R, colony-stimulating factor 1 receptor; HMGB1, the high-mobility group box 1*.

### Microglia and Aβ

As a hallmark of AD pathology, Aβ has attracted extensive attention and has been intensively investigated since it was first identified by Alois Alzheimer (Alzheimer et al., 1995). Although converging evidence implicates that microglia are pivotal players in Aβ plaque formation, the underlying mechanisms by which microglia modulate plaque dynamics remain largely unknown. Specifically, whether the failure of Aβ clearance is related to the functional state (activation or senescence) of microglia has been inconsistent across various studies. Traditionally, it was believed that microglia help clear Aβ aggregation *via* phagocytosis (Pan et al., 2011; Hellwig et al., 2015; Yin et al., 2017). Ablation of microglia in wild-type (WT) organotypic hippocampal slice cultures promotes accumulation of Aβ deposits; however, replenishment with cultured primary microglia can deplete plaque formation by promoting the digestion of Aβ by phagocytosis and its subsequent degradation (Hellwig et al., 2015). Moreover, different Aβ conformations may affect microglial phagocytosis. An *in vitro* study revealed that it was not the early-stage component of (oligomeric) Aβ but the terminal (fibrillar) Aβ that enhanced the phagocytic activity of microglia (Pan et al., 2011). Oligomeric Aβ_1–42_ inhibited fibrillar Aβ_1–42_-elicited microglial phagocytosis, which might be ascribed to increased levels of inflammatory mediators. Anti-inflammatory agents and antioxidants rescued the compromised microglial phagocytosis caused by oligomeric Aβ_1–42_. With the advance of aging, the phagocytic capacity of microglia is impaired (Safaiyan et al., 2016; Pluvinage et al., 2019). Microglial phagocytosis occurs in early onset AD brains rather than in late-onset AD (LOAD) brains (Yin et al., 2017). Consistent with these findings, microglia-depleted organotypic hippocampal slice cultures replenished with forebrain microglia from adult 5xFAD mice were found to display obvious Aβ plaque formation (Hellwig et al., 2015), suggesting that healthy microglia might play a neuroprotective role compared to dystrophic or senescent microglia. However, the exhaustion of phagocytosis in microglia was probably due to the chronic stimuli of plaque load or high Aβ levels and not just to aging or the genetic background of AD transgenic mice. Indeed, microglia isolated from the cerebellum (an Aβ-free brain area) of juvenile or young adult WT or 5xFAD mice exhibited no differences in the formation of Aβ plaques (Hellwig et al., 2015). Additionally, this explanation makes it easier to understand why microglial ablation had no effect on Aβ plaque load in either young or aged AD transgenic mice (Grathwohl et al., 2009).

In accordance with these findings, we learned that microglia are fully capable of clearing Aβ in a favorable environment. However, the underlying molecular mechanisms of recognition, internalization, and clearance of Aβ by microglia remain unclear. Scavenger receptors (SRs) are defined as cell surface receptors that eliminate degraded or harmful substances (PrabhuDas et al., 2017). SRs are highly expressed on the surface of phagocytes, such as macrophages and microglia, and share the ability to bind many ligands, including Aβ. SRs were first identified in 1979 as macrophage receptors responsible for the uptake of modified low-density lipoprotein (Goldstein et al., 1979). A number of factors have been shown to regulate SR expression. In addition to modified or oxidized low-density lipoproteins, cytokines (Bottalico et al., 1991; Geng and Hansson, 1992; de Villiers et al., 1994; Hsu et al., 1996; Yesner et al., 1996; Khovidhunkit et al., 2001; Murphy et al., 2005; Hashizume and Mihara, 2012), including macrophage colony-stimulating factor (M-CSF), tumor necrosis factor (TNF)-α, IFN-γ, transforming growth factor (TGF)-β1, and IL-1, IL-4, and IL-6, hormones (testosterone and estrogen; Langer et al., 2002; Srivastava, 2003), cell differentiation transcription factors [peroxisome proliferator-activated receptors (PPAR) α and γ; Chinetti et al., 2000], antioxidants (N-acetylcysteine, kaempferol; Svensson et al., 2002; Li et al., 2013), endotoxin (LPS; Khovidhunkit et al., 2001; Baranova et al., 2002; Park et al., 2012), and bacteria or dead or apoptotic cells (Grolleau et al., 2003), also affect SRs expression.

Currently, the SR family is classified into 10 classes, named SR-A–SR-J (PrabhuDas et al., 2017). In the brains of patients with AD, SRs are prominent in activated microglia surrounding senile plaques (Christie et al., 1996). In an *in vitro* study, SRs in microglia were found to mediate the binding and internalization of Aβ microaggregates (Paresce et al., 1996). SR-A plays an important role in the adhesion of microglia to Aβ fibrils (El Khoury et al., 1998) and is considered the main receptor for the uptake of fibrillar Aβ (Chung et al., 2001). Based on short-hairpin RNA screening, Frenkel et al. (2013) identified the class A1 scavenger receptors (Scara1) as the primary phagocytic receptors responsible for the clearance of the soluble forms of Aβ. *Scara1* deficiency resulted in an approximately 50% reduction in Aβ uptake by the microglia and led to a significant increase in Aβ deposition in amyloid precursor protein (APP)/presenilin 1 (PS1) transgenic mice (Frenkel et al., 2013). In SR-A knockout mice, the ability of microglia to take up fibrillar Aβ was found to be reduced by 60% compared to that in WT mice (Chung et al., 2001). Moreover, fibrillar Aβ uptake in SR-A-knockout mice was competed by other SR ligands. Based on these results, other SRs, including SR-B, may also be involved in the uptake of Aβ by microglia. CD36, the prototype SR-B, has been widely studied in the uptake of Aβ and microglial-induced pro-inflammatory events, which appear to play a dichotomous role in AD progression. CD36 is an important nexus for Aβ recognition by microglia. Microinjection of fibrillar Aβ causes significantly less microglial recruitment when in injected into CD36^−/−^ mice than into CD36^+/+^ mice (El Khoury et al., 2003), which suggests that microglial recruitment for Aβ deposition is dependent on CD36. Furthermore, TREM2 (triggering receptor expressed on myeloid cells 2)-mediated Aβ phagocytosis also requires CD36 expression (Kim et al., 2017). However, CD36 is involved in the pro-inflammatory events associated with AD. In activated microglia, CD36 mediates the release of neurotoxic reactive oxygen species (ROS), pro-inflammatory cytokines, and chemokines. These pro-inflammatory substances, induced by fibrillar Aβ, were found to be reduced in microglia isolated from CD36-deficient mice (El Khoury et al., 2003). Inhibition of the CD36-Aβ interaction can also block the microglial immune response to Aβ (Doens et al., 2017). Moreover, CD36 is considered an absolute requirement for neurovascular dysfunction and cerebrovascular oxidative stress induced by Aβ (Park et al., 2011). Although studies on CD36 report incongruent results, they contribute to the exploration of new immunomodulatory agents for AD therapeutics. The receptor for advanced glycation end products (RAGE) is also a member of the SR family and belongs to the class J SR group (PrabhuDas et al., 2017). RAGE-immunoreactive microglia have been found to be increased in either Aβ-rich regions of AD brains or in *in vitro* cell models (Lue et al., 2001). RAGE-Aβ interaction plays an important role in the chemotactic response of microglia to Aβ deposits. RAGE blockade leads to reduced microglial migration toward Aβ deposits (Lue et al., 2001). More recently, numerous studies have reported the pathogenic role of RAGE in AD progression, which promotes the release of pro-inflammatory mediators by activated microglia (Fang et al., 2010; Deane et al., 2012; Cathrine C et al., 2020). A potent multimodal RAGE blocker effectively inhibited the microglial activation and neuroinflammatory response to Aβ and improved the cognitive performance in AD transgenic mice (Deane et al., 2012). Based on an extensive literature review, SR-A appears to be the most important SRs involved in Aβ clearance. Although CD36 and RAGE also play a role in Aβ clearance, these SRs appear to be double-edged swords in controlling the interaction between the microglia and Aβ as they may play a role in both AD progression and prevention. This leads to the thought-provoking question of whether the dysregulation of SRs is causative of AD, or a consequence of its pathological progression. It is well-known that Aβ deposition is a hallmark of AD pathology, which is an early pathological event in AD progression. In the early stages of AD, microglia possess the ability to clear Aβ with the help of SRs. The potential mechanisms for the involvement of SRs in Aβ clearance by the microglia may be the recruitment of microglia to Aβ deposition sites and the mediation of microglial adhesion to Aβ-coated surfaces (El Khoury et al., 1996; Cornejo and von Bernhardi, 2013). Several studies have reported that the increase in Aβ deposition is accompanied by corresponding changes in some SRs, including the activation of SR-A (Zhang et al., 2014), the increased expression of SR-B type I (SR-BI; Thanopoulou et al., 2010; Eugenín et al., 2016), and complicated changes in CD36 levels (Coraci et al., 2002; Ricciarelli et al., 2004). In this sense, the dysregulation of SRs may be a consequence of AD progression. However, whether the dysregulated expression of SRs is a major instigator of AD pathological progression is unclear. Therefore, future studies should elucidate their roles in AD pathology.

With the advent of genome-wide association studies (GWAS), a novel set of AD risk genes associated with microglial function have been identified, including *Apolipoprotein E* (*APOE*)*, Complement receptor 1* (*CR1*), *TREM2*, *CD33*, *Membrane-spanning 4-domain subfamily A* (*MS4A*), *ATP-binding cassette (ABC) transporter A7* (*ABCA7*), and* Inositol polyphosphate-5-phosphatase* (*INPP5D*)*/Src homology 2-containing inositol-5’-phosphatase 1* (*SHIP1*; Malik et al., 2015; Efthymiou and Goate, 2017; McQuade and Blurton-Jones, 2019; Nguyen et al., 2020). These genes confer a risk of AD by modulating the activation of microglia, which is determined by the promotion of phagocytosis and the release of inflammatory mediators. Compelling evidence from *in vivo* and *in vitro* studies has demonstrated that ApoE plays an important role in regulating the CNS inflammatory and immune response (Lynch et al., 2003; Pocivavsek et al., 2009). However, the molecular mechanisms underlying ApoE-dependent immunomodulation in AD remain unclear. Microglia are thought to play a central role in regulating the interaction between ApoE and amyloid plaque pathology. ApoE deficiency can influence microglial responses to amyloid plaques. The number of microglia located near Aβ plaques was found to be significantly decreased in *APOE*^−/−^; APP/ PS1 and *APOE*^−/−^; APP/PS1ΔE9 transgenic mice (Ulrich et al., 2018). However, a study of AD human tissues revealed that the* APOE ε4* genotype is related to a dampened microglial response to amyloid plaques (Nguyen et al., 2020). A possible explanation for the discrepancies between these studies is that the* APOE* gene includes three different alleles: *ε2*, *ε3*, and *ε4*. The *ε4* allele raises AD risk, whereas the *ε2* allele lowers AD risk. Moreover, ApoE is an anti-inflammatory agent (Malik et al., 2015); thus, ApoE deficiency reduces the microglial response to Aβ and amplifies the inflammatory cascade. In addition to *APOE*, *CR1* is also an important genetic susceptibility locus for LOAD. There is growing evidence that some single-nucleotide polymorphisms (SNPs) of CR1 confer AD risk and are associated with Aβ accumulation (Lambert et al., 2009; Zhu et al., 2015, 2020). The potential role of CR1 in AD pathogenesis may be related to microglia-mediated Aβ phagocytosis. CR1 is presented on microglia, and its expression is increased upon the activation of microglia. Some studies have identified that blocking CR1 leads to a decrease in the capacity of the microglia to phagocytose Aβ (Crehan et al., 2013).

Although TREM2 has been extensively studied as a microglial surface receptor, whether its role in AD pathology is protective, detrimental, or inconsequential is still a subject of debate. GWAS have identified the R47H variant of *TREM2* as a risk factor for AD associated with a decrease in amyloid-responsive microglia in the human brain (Ulland et al., 2017; Nguyen et al., 2020). The expression of *TREM2* was found to be increased in microglia in plaque-loaded brain regions of AD transgenic mice (Frank et al., 2008), implying that TREM2 is a prominent player in AD progression. Mounting evidence reveals that TREM2 can sustain microglial responses to Aβ deposition and promote microglial phagocytosis (Wang et al., 2015; Ulland et al., 2017; Ruganzu et al., 2021). In *TREM2*^−/−^5xFAD mice, microglia failed to cluster around Aβ plaques, resulting in less effective Aβ phagocytosis and clearance (Wang et al., 2015; Ulland et al., 2017), a result that has been replicated in AD patients carrying TREM2 risk variants (Ulland et al., 2017). Why does TREM2 deficiency impair microglial responses to Aβ accumulation? The potential explanation given by Colonna and colleagues was that defective TREM2 function deflects the mammalian target of rapamycin signals, which are responsible for energy and anabolic metabolism; therefore, autophagic flux is considered an effective compensatory response to metabolic defects (Ulland et al., 2017). The increase in autophagic-like vesicles in microglia led to the death of microglia and a decrease in the number of microglia. Thus, microglia are unable to efficiently aggregate around Aβ plaques due to insufficient energy supply and dysfunction. CD33 is thought to act in the opposite direction to TREM2, as it can reduce Aβ phagocytosis and increase the risk of AD (Bradshaw et al., 2013; Griciuc et al., 2013; Yin et al., 2017).

Although *ABCA7*, *MS4A*, and *INPP5D* are poorly characterized, the critical role of these genes in the phagocytosis of Aβ by the microglia has been identified by a few studies. *MS4A* cluster variants have been reported to modulate the risk of AD in several GWAS (Mao et al., 2015; Chang et al., 2019). MS4A modulates soluble TREM2 production and interacts with APOE, which appears to be an important modulator in AD pathogenesis (Ma et al., 2015; Chang et al., 2019; Deming et al., 2019). ABCA7, a member of the ABC subfamily-A transporters, is strongly expressed in the brain and has been detected in the microglia (Kim et al., 2006). In addition to lipid transport, ABCA7 has been implicated in the microglial Aβ clearance pathway. ABCA7 deficiency aggravates AD pathogenesis by impairing proper microglial responses to Aβ aggregates (Kim W. S. et al., 2013; Fu et al., 2016; Aikawa et al., 2019). *INPP5D* is also a microglia-related gene, involved in LOAD. Studies have reported that *INPP5D* expression is upregulated with the progression of AD and is detected mainly in the plaque-associated microglia, implicating a deleterious role of increased *INPP5D* expression in AD (Tsai et al., 2021). Consequently, targeting these AD-related risk genes may offer new avenues for AD treatment.

As mentioned above, the interplay between microglia and Aβ is intricate. How do microglia access Aβ plaques? What are the molecular mechanisms of recognition, internalization, and clearance of Aβ by microglia? Although a multitude of microglia-associated molecules, including genes and SRs, have been extensively investigated, the precise effects of these molecules remain controversial. On these bases, further experiments are needed to gain clarity; after all, from a therapeutic standpoint, the prospect of targeting microglia-Aβ interactions is encouraging.

### Microglia and Neuroinflammation

Inflammation is considered a prominent factor in triggering the onset of AD that may occur even before amyloid deposition (Heneka et al., 2005a), and plays an important role in AD pathology. Interestingly, Aβ deposits, chronic microglial activation, and microglial inflammatory mediators are considered key mechanisms underlying the vicious cycle of inflammation in the course of AD. As mentioned above, some AD risk genes and dysregulated SRs are involved in the release of inflammatory mediators from microglia, exacerbating neuroinflammation in AD. Moderately activated microglia manifest neuroprotective effects in the early stages of AD by phagocytosis of Aβ deposits. However, microglia do not rapidly clear Aβ. Therefore, Aβ aggregates to form extracellular plaques and continue to stimulate microglial activation. Over-activated microglia continuously release inflammatory mediators such as TNF-α, IL-6, IL-1β, nitric oxide (NO), and monocyte chemoattractant protein-1 (MCP-1; Ishizuka et al., 1997; Akiyama et al., 2000; Fang et al., 2010; Magni et al., 2012), which eventually leads to the enduring chronic neuroinflammatory environment of AD. As an endogenously synthesized free radical, NO appears to play a prominent role in microglia-mediated inflammatory events. NO is synthesized by three distinct nitric oxide synthases: the inducible NO synthase (iNOS), the neuronal NO synthase (nNOS), and the endothelial NO synthase (eNOS; Balez and Ooi, 2016). To our knowledge, eNOS and nNOS may be constitutively expressed in the normal brain; however, the increased expression of iNOS in the CNS is considered pathological (Fernandez et al., 2010). In AD pathogenesis, iNOS is principally produced by reactive microglia and astrocytes during Aβ-elicited inflammatory responses, which then trigger the release of high quantities of NO (Asiimwe et al., 2016). Additionally, iNOS deficiency induced by genetic ablation or pharmacologic inhibition can reduce the cerebral plaque burden and inhibit microgliosis and astrocytosis (Nathan et al., 2005). The contextual link between NO and AD mainly focuses on NO-induced oxidative stress, which amplifies the inflammatory and immune responses in AD pathology *via* lipid peroxidation and protein S-nitrosylation (Asiimwe et al., 2016). NO interacts with free radical superoxide to form highly reactive nitrogen species, such as peroxynitrite. Peroxynitrite oxidizes a series of molecules, including lipids, proteins, and DNA, which aggravates the oxidative damage to the membrane polyunsaturated fatty acids; damages respiratory enzymes, resulting in a decrease in ATP production; and causes structural and functional DNA impairment. Noxious NO levels also impair the mitochondrial dynamic network through nitrosylating dynamin-related protein 1, leading to mitochondrial fragmentation (Cho et al., 2009; Nakamura and Lipton, 2011).

The role of the NOD-like receptor family pyrin domain-containing 3 (NLRP3) inflammasome in AD has been subject of thorough investigation. *In vivo* or *in vitro* models of AD confirm that microglial inflammasome activation is a potent contributor to AD pathology (Heneka et al., 2013; Gustin et al., 2015). The inflammasome controls caspase-1 activity; in-turn, activated caspase-1 regulates the maturation and secretion of IL-1β. Microglia can express NLRP3 inflammasome-related components, including the NLRP3 protein, apoptotic speck-containing protein with a caspase recruitment domain (ASC), and caspase-1 (Gustin et al., 2015). After priming with LPS, NLRP3 expression is further upregulated in microglia, accompanied by the cleavage and secretion of IL-1β and IL-18, in an NLRP3 inflammasome-dependent manner (Gustin et al., 2015; Freeman et al., 2017). Golenbock and colleagues reported that the amount of caspase-1 in the hippocampus or frontal cortex was substantially increased in patients with AD and MCI compared with controls (Heneka et al., 2013), which implicated chronic inflammasome activation. Increased caspase-1 expression was also found to occur in APP/PS1 transgenic mice. Importantly, NLRP3 or caspase-1 deficiency could protect mice from spatial memory impairment, improve neuronal function, reset microglial phagocytosis, and even reduce Aβ deposition (Heneka et al., 2013). Collectively, Aβ-induced microglial NLRP3 inflammasome activation disrupts Aβ phagocytosis and clearance and amplifies its destructive effects through a vicious cycle of the inflammatory cascade.

Although over-activated microglia are an important contributor to uncontrolled neuroinflammation in AD pathogenesis, during the early stages of the disease, microglia also share the ability to limit and balance inflammatory responses *via* endogenous anti-inflammatory systems. Suppressors of cytokine signaling (SOCS) proteins are important players in these processes. The SOCS family of proteins is composed of eight protein members with similar structures and properties, named SOCS 1–7 and cytokine-inducible SH2-containing protein (CIS). These proteins act as negative feedback regulators of the Janus kinase (JAK)/signal transducer and activator of transcription (STAT), nuclear factor-κB (NF-κB), and toll-like receptor 4 (TLR4) pathways (Yoshimura et al., 2005). Evidence from studies on autopsy brain tissues and on cellular and transgenic mouse models has revealed that SOCS proteins, especially SOCS1 and SOCS3, play a role in modulating microglial inflammatory signaling in AD (Cianciulli et al., 2017; Iwahara et al., 2017; Ruganzu et al., 2021). Cianciulli et al. reported that all *SOCS* mRNA and proteins could be constitutively detected in AD and non-dementia brain samples, and increased expression of some key SOCS genes was observed in vulnerable areas of AD patients (Cianciulli et al., 2017). Consistently, both* SOCS1* and *SOCS3* mRNA levels were increased in the Aβ-stimulated primary microglia, and obviously increased *SOCS3* mRNA was found in AD transgenic mice (Iwahara et al., 2017). However, in contrast to these results, reduced SOCS1 and SOCS3 were found in APP/PS1 mice, accompanied by increased phosphorylation of JAK2, STAT1, and STAT3 (Ruganzu et al., 2021), suggesting that the SOCS signaling pathway is impaired in AD progression.

Taken together, these data suggest that chronic microglial activation plays a detrimental role in the pathogenesis of AD. In addition to the functional status of the microglia, we also need to focus on the timing of neuroinflammation in AD progression. In particular, microglia-associated endogenous anti-inflammatory systems (i.e., SOCS) play a protective role by balancing the inflammatory response during the early stages of the disease. Therefore, it is important to identify the shift in mechanism during the pathological stages of the disease. On this basis, appropriate inhibition of microglial activity or inflammation-targeted therapeutic approaches may uncover effective therapies to attenuate the pathological progression of AD.

### Microglia and Tau Pathology

Tauopathies, which are characterized by the pathological aggregation of tau, are considered another key hallmark of AD pathology. Extensive research has shown that microglial dysfunction is also associated with tau pathology and that they may have a bidirectional causal relationship in AD progression. Functionally, microglia have the capacity to phagocytose the pathological aggregation of tau (Luo et al., 2015; Bolos et al., 2016; Brelstaff et al., 2018; Hopp et al., 2018). According to a compelling study, microglia colocalized with NFTs in AD postmortem brains and internalized aggregated tau *in vivo* and *in vitro* (Bolos et al., 2016). Microglia can also internalize and degrade hyper-phosphorylated tau isolated from the brain sections of postmortem AD patients or P301S transgenic mice (Luo et al., 2015). Furthermore, the anti-tau monoclonal antibody promoted tau internalization and degradation by the microglia, a process that is dependent on the Fc effector (Luo et al., 2015). Consistently, another study reported that activated microglia engulfed neurons with insoluble tau inclusions in a manner dependent on opsonin milk-fat-globule EGF-factor-8 (MFGE8) and NO production (Brelstaff et al., 2018).

However, aberrant microglial activation also facilitates tau pathology (Bhaskar et al., 2010; Cho et al., 2011; Maphis et al., 2015; Clayton et al., 2021). CX3C chemokine ligand 1 (CX3CL1), also known as fractalkine, is expressed in neurons, and its sole ligand, CX3C chemokine receptor 1 (CX3CR1), is almost exclusively expressed in the microglia (Perea et al., 2018). The CX3CL1/CX3CR1 axis plays an important role in the neuron-microglia crosstalk. Studies suggest that CX3CR1 plays a pivotal role in microglia-mediated tau pathology (Bhaskar et al., 2010; Cho et al., 2011). The levels of both CX3CL1 and CX3CR1 were found to be significantly lower in the hippocampus and frontal cortices of AD brains than in those of controls (Cho et al., 2011), indicating an impairment of CX3CL1/CX3CR1 signaling in AD. Furthermore, increased tau phosphorylation was observed in AD transgenic mice lacking CX3CR1 (Cho et al., 2011). Consistently, altered microglial activation has been observed in tauopathy mice (Bhaskar et al., 2010). Systemic LPS administration to tauopathy mice evidently enhances microtubule-associated protein tau (MAPT) phosphorylation at theSer202 and Thr231 sites (Bhaskar et al., 2010), suggesting that enhanced microglial activation is critical in exacerbating MAPT phosphorylation. Moreover, the loss of *Cx3cr1* causes further increase in microglial activation and MAPT phosphorylation in tauopathy mice. Additionally, variants in microglial-expressed genes, including *CSF1R* (Mancuso et al., 2019a; Clayton et al., 2021), *APOE* (Shi et al., 2017)*, and TREM2* (Leyns et al., 2019), are also involved in tau-induced neurodegeneration. Targeting these microglial receptors may pave the way for exploring immunotherapeutic strategies to attenuate tau pathology in AD.

However, there is an incongruent opinion from Bechmann and colleagues (Streit et al., 2009). Given that AD is inextricably associated with aging, it is not surprising that senescent microglia worsen AD pathology. Bechmann et al. revealed that senescent microglia, but not activated microglia, contribute to tau pathology. Baker and colleagues also reported that senescent microglia play a pathogenic role in tau-mediated pathology (Bussian et al., 2018). Senescent microglia have been found in *tau MAPT P301S PS19* (*PS19*) mice. As evidenced by an animal model based on the crossing of* INK-ATTAC* transgenic mice (*ATTAC*) and the *PS19* strain to generate *PS19*;*ATTAC* mice, AP20187 administration results in the elimination of gliosis and glial cell senescence. Moreover, AP20187-treated *PS19*;*ATTAC* mice exhibit attenuated tau phosphorylation and improved cognitive function, suggesting that depletion of senescent microglia could protect against tau-dependent pathology.

In addition to the internalization and degradation of hyper-phosphorylated tau proteins, the microglia also participate in tau propagation (Asai et al., 2015; Maphis et al., 2015; Clayton et al., 2021). It is well established that tau proteins share prion-like spreading properties, by active neuron-to-neuron transfer or by contaminating secondary cells through a seeding mechanism (Dujardin and Hyman, 2019). However, the role of microglia in tau dissemination is still a matter of debate. According to an *in viv*o study, activated microglia spread pathologic tau from CA1 neurons to the subiculum in a time-dependent manner (Maphis et al., 2015). AT180 (a MAPT antibody)-positive neurons were found to be evident in the CA1 region of the hippocampus in 2-month-old hTau (expressing a human tau mutation) *Cx3cr1*^−/−^ mice, while few AT180 positive neurons were found in the subiculum. By 24 months of age, numerous AT180 positive neurons were found in the subiculum, accompanied by most activated microglia. Another study published at the same time revealed that microglia indeed promote tau transmission (phagocytosis and secretion) through anatomically connected neurons (Asai et al., 2015). Microglial depletion inhibits tau propagation from the entorhinal cortex (EC) to the dentate gyrus (DG). Importantly, extracellular vesicles play a critical role in microglia-mediated tau propagation from neuron to neuron. That study further reported that tau transduction was dependent on exosome secretion, as suppression of exosome synthesis reduces microglia-mediated tau secretion (Asai et al., 2015). Ikezu and colleagues also confirmed that microglia-derived extracellular vesicles containing phosphorylated tau accelerated tau propagation in AD transgenic mice (Clayton et al., 2021).

### Microglia and Mitophagy

Mitochondria are known as the sites of ATP production and are involved in the energy metabolic pathways of neurons and glial cells, playing an important role in their survival and diverse functions. Accumulating evidence suggests that AD involves damaged mitochondria (Fang et al., 2019), which leads to bioenergetic deficits, triggers lipid peroxidation, releases large amounts of ROS, and further promotes the accumulation of Aβ and hyper-phosphorylated tau. Accumulation of damaged mitochondria suggestive of impaired mitophagy has been found in AD patients as well as in animal and cellular AD models, which suggests impaired mitophagy. The molecular and cellular mechanisms underlying mitophagy impairment in AD remain unclear. Recent advances in the understanding of compromised mitophagy in AD have revealed the involvement of microglia (Lei et al., 2018; Fang et al., 2019). Microglia are best-known as CNS phagocytes responsible for Aβ clearance. However, dysfunctional microglia are involved in the AD microenvironment, leading to impaired phagocytic function and defective mitophagy in the microglia. Microglial mitophagy is decreased in AD mice compared with WT-mice (Fang et al., 2019). Mitophagy stimulation can enhance the clearance of Aβ plaques by microglia, as well as increase microglial population, improve mitochondrial damage, and decrease neuroinflammation (Fang et al., 2019; Ahmed et al., 2021). The potential explanations for mitophagy stimulation to improve microglial phagocytosis are the restoration of ATP supply and common phagocytic mechanisms between the two; however, which signaling pathways are involved in the mitophagy profiles of microglia is still unknown. Xia and colleagues found that the high-mobility group box 1 (HMGB1)/RAGE axis mediates compromised mitophagy flux in microglia (Zhang et al., 2020). In stressed microglia, the expression of HMGB1 and RAGE was upregulated and was accompanied by the accumulation of damaged mitochondria. The HMGB1/RAGE axis plays an important role in blocking late-stage mitophagy in the microglia, which can be reversed by RAGE deficiency. Mitophagy-related downstream events were found to be improved in RAGE^−/−^ microglia, including increased p62 degradation and elevated acidifying lysosomes. Furthermore, facilitation of mitophagy flux inhibited the proinflammatory phenotypic switch of microglia. Inflammasome activation also plays a role in the blockade of mitophagy in the microglia. Following an exposure to the immunodeficiency virus type-1 (HIV-1) long terminal repeat region (ssRNA40), the microglia exhibited increased mitochondrial aggregates and blocked autophagy/mitophagy. Defective autophagy/mitophagy abolished the negative regulation of the NLRP3 inflammasome; therefore, NLRP3 activity is increased in HIV-1 ssRNA40-stimulated microglia (Rawat et al., 2019). NLRP3 activation increases the release of proinflammatory cytokines and compromises the functional and structural integrity of mitochondria, causing damage to mitochondrial aggregates in microglia. Taken together, NLRP3 activation and defective mitophagy may interact in a bidirectional manner. From the above discussion, we conclude that targeting mitophagy not only inhibits the neuroinflammation triggered by defective microglia or mitochondria but also enhances the microglial phagocytic activity to maintain the microenvironmental homeostasis.

## Targeting Microglia for AD Treatment

Although many efforts are underway to help halt or reverse the underlying pathology of this multifactorial and complex disease, few tangible results have been translated into clinical treatment. Based on the aforementioned discussion, we noted that microglia are important mediators in the development and progression of AD. The functional states of microglia play a particularly important role in health and disease, as dysfunctional microglia lose their phagocytic capacity and subsequently trigger an inflammatory cascade that exacerbates AD pathology. Thus, microglia-targeted therapies may provide a novel therapeutic avenue for treating AD (Table 2).

**Table 2:** Microglia-targeted therapies in AD.

Target	Mechanisms	Reagents/Interventions	References
Microglial Modification Therapeutics	Depletion of dysfunctional microglia	PLX3397; PLX5622	Sosna et al. (2018) and Spangenberg et al. (2019)
		Deletion of a *Csf1r* enhancer: the *fms*-intronic regulatory element (FIRE)	Rojo et al. (2019)
	Replenishment of healthy microglia	Stem cell transplantation	Muffat et al. (2016); Douvaras et al. (2017); Haenseler et al. (2017); Mancuso et al. (2019b); Kim et al. (2012); Kim K.-S. et al. (2013); Lee et al. (2015); and Kim et al. (2015)
Targeting Microglial Immunoreceptors	Enhancement of TREM2 activity to increase microglial responses to Aβ	AL002; AL002a; AL002c; Monoclonal antibody 4D9	Cheng et al. (2018); Price et al. (2020); Wang et al. (2020); and Schlepckow et al. (2020)
	Inhibition of CD33 activity to increase Aβ phagocytosis	P22; Lintuzumab	Zhang et al. (2016) and Miles et al. (2019)
Targeting Inflammatory Response in Microglia	Improvement of microglia over-activation and inhibition of microglia-associated inflammatory events	Ibuprofen; Pioglitazone; MCC950; JC-124; Minocycline; Edaravone; Oxidized ATP; Brilliant blue G; Nimodipine	Yan et al. (2003); McLarnon et al. (2006); Ryu and McLarnon (2008); Geldmacher et al. (2011); Sato et al. (2011); Wilkinson et al. (2012); Jiao et al. (2015); Yang et al. (2015); Wang et al. (2017); Parikh et al. (2018); Yin et al. (2018); Chiozzi et al. (2019); Garcez et al. (2019); and Lučiūnaitė et al. (2020)

### Microglial Modification Therapeutics

It is well established that microglia play a dual role in AD progression. Early microglial activations exert neuroprotective effects by promoting Aβ clearance and share endogenous anti-inflammatory activity; however, when the Aβ load increases over the course of AD, over-activated microglia acquire a pro-inflammatory phenotype and in turn facilitate Aβ accumulation and accelerate AD pathology. Therefore, eliminating dysfunctional microglia or replenishing healthy microglia may provide novel treatment options for AD.

Several studies have shown that microglia depletion yields tangible results in AD transgenic mouse models (Asai et al., 2015; Sosna et al., 2018; Spangenberg et al., 2019; Casali et al., 2020). CSF1R is indispensable for the survival and development of microglia; thus, continued administration of CSF1R inhibitors is a non-invasive and effective approach to specifically ablate the microglia, which has been adopted in numerous studies. Glabe and colleagues reported that after a 3-month administration of the selective CSF1R inhibitor PLX3397 to 5XFAD mice, the number of microglia was reduced by approximately 70–80% (Sosna et al., 2018). Long-term treatment of 5XFAD transgenic mice with PLX3397 was found to improve cognitive impairment and amyloid pathology in AD-affected brain regions (Sosna et al., 2018). Moreover, *in vivo* PLX3397-induced microglial depletion was also found to suppress tau propagation (Asai et al., 2015), thereby exerting a neuroprotective effect. Consistently, another CSF1R inhibitor, PLX5622, which has good oral bioavailability and brain-penetration properties, was reported to prevent plaque formation when administered continuously (from 1.5 months of age until 4 or 7 months of age) to 5XFAD mice (Spangenberg et al., 2019). A subsequent study by Landreth and colleagues also found a reduction in overall plaque load by blocking the CSF1R (Casali et al., 2020). However, this study provided a refined understanding of microglial ablation in plaque deposition kinetics during the pathological progression of 5XFAD transgenic mice (4 months old), as it revealed that microglial ablation leads to the alteration of plaque morphology from compact to diffuse. That study demonstrated that microglia play an important role in limiting plaque expansion, even during the peak period of plaque formation. Apart from the pharmacological interventions mentioned above, genetic techniques can also be used to ablate the microglia. Deletion of a *Csf1r* enhancer, the *fms*-intronic regulatory element (FIRE), can produce microglia-deficient animal models (Rojo et al., 2019). Cre-mediated recombination led to the specific expression of the diphtheria toxin receptor by microglia under the control of *the Cx3cr1* promoter. When treated with the diphtheria toxin, microglia were specifically ablated by approximately 80% (Bruttger et al., 2015). Genetic interventions may provide a more intuitive approach to explore physiological and immunological microglial functions in AD. Although microglial ablation provides a new direction for disease-modifying therapies in AD, many factors need to be discussed and evaluated, such as the functional status of the microglia at specific stages of AD and the precise timing of microglial ablation; thus, major challenges must be addressed before these therapies are clinically available.

Similarly, from a therapeutic standpoint, the replenishment of healthy microglia may also be advantageous in ameliorating AD pathologies. In line with this, transplanted stem cell therapy appears to have the potential to achieve the therapeutic goal of repairing the dysfunctional microglia in the course of AD. Recently, microglia-like cells have been successfully derived from human stem cells, including induced pluripotent stem (iPS) cells and embryonic stem (ES) cells (Muffat et al., 2016; Douvaras et al., 2017). Stem cell-derived microglia share a similar signature with purified human fetal microglia and exhibit efficient phagocytosis and a rapid response to detrimental stimuli (Muffat et al., 2016; Douvaras et al., 2017; Haenseler et al., 2017). Furthermore, stem cell-derived microglia can survive and integrate into the mouse brain after transplantation (Mancuso et al., 2019b). Accumulating evidence from *in vivo* studies has confirmed the neuroprotective effects of transplanted stem cells. The use of stem cell therapies in AD transgenic mice facilitate improvement of memory deficits and related neuropathology (Kim et al., 2012; Kim K.-S. et al., 2013; Kim et al., 2015; Lee et al., 2015).

### Targeting Microglial Immunoreceptors

Owing to well-powered GWAS, a series of AD risk genes have been identified, some of which are immunoreceptors expressed in the microglia. Among these various microglial receptors, *TREM2* and *CD33* have been extensively investigated because they are the top-ranked AD risk genes. As described above, the precise role of *TREM2* in AD pathology remains a matter of debate. Increasing evidence suggests that *TREM2* is required for microglial response to Aβ, and that *TREM2* deficiency aggravates AD pathology, causing decreased Aβ phagocytosis and clearance (Wang et al., 2015; Ulland et al., 2017; Ruganzu et al., 2021). Accordingly, a growing body of studies have aimed to explore the effects of TREM2 agonists on AD pathology. Several TREM2 agonistic antibodies, including antibody 1 (Cheng et al., 2018), antibody 2 (Cheng et al., 2018), AL002c (Wang et al., 2020), and AL002a (Price et al., 2020), have been found to exhibit neuroprotective effects by ameliorating Aβ pathology and increasing microglial responses to Aβ. Notably, the clinical variant of AL002c, AL002, was well tolerated in a human phase 1 clinical trial (Wang et al., 2020). Another recent study revealed a new way to enhance TREM2 activity by reducing proteolytic shedding (Schlepckow et al., 2020). Monoclonal antibody 4D9 reduced the shedding of TREM2 and stabilized its expression on the cell surface *via* bivalent binding. Importantly, 4D9 enhanced the microglial response to Aβ and increased Aβ phagocytosis *in vitro* and *in vivo* (Schlepckow et al., 2020).

It is clear that polymorphisms of the *CD33* gene are involved in AD susceptibility and pathology, and the CD33 sialic acid-binding domain may be a potential action site for CD33-mediated suppression of Aβ phagocytosis (Zhao, 2019). Thus, targeting the sialic acid-binding domain may represent a promising approach for the treatment of AD. Parker and colleagues identified a novel sialic acid-based ligand, P22, which is a subtype-selective sialic acid mimetic (Miles et al., 2019). P22 was found to increase Aβ phagocytosis when it was conjugated to microparticles in a CD33-dependent manner. CD33 inhibitory antibodies may provide another means of resistance to the neurotoxic effects of CD33. Based on a database search, CD33 was identified as the strongest candidate for potential anti-AD targets (Zhang et al., 2016). That research article pointed out that several existing CD33 inhibitory antibodies may be repurposed as anti-AD therapies. In particular, lintuzumab, which is currently used for treating acute myelogenous leukemia, may be a viable candidate for treating AD. Further experiments are needed to evaluate and validate the feasibility of using this antibody for the treatment of AD.

### Targeting Inflammatory Response in Microglia

The microglia are involved in neuroinflammation through a series of molecular interactions and intercellular communications. Imbalanced microglial activation or insufficient microglial phagocytic capacity can accelerate the accumulation of extracellular Aβ and intracellular NFTs and release large amounts of pro-inflammatory cytokines, chemokines, and ROS. Therefore, normalizing the over-activation of microglia and enhancing their phagocytotic capabilities may mitigate the inflammatory responses in AD. Non-steroidal anti-inflammatory drugs (NSAIDs) have been extensively explored for the treatment of AD. Chronic NSAID treatment can ameliorate the pathology of AD. Ibuprofen, the most commonly used NSAID, can reduce Aβ plaque load, microglial activation, and proinflammatory cytokine levels *in vivo* and *in vitro* (Lim et al., 2000; Yan et al., 2003; Heneka et al., 2005b; Wilkinson et al., 2012). The potential molecular mechanism behind NSAID-mediated neuroprotective effects appears to be associated with PPARγ. Upon activation by NSAIDs, PPARγ exerts transcriptional regulation by repressing the expression of pro-inflammatory genes (Heneka et al., 2005b). Thus, the PPARγ agonist pioglitazone has been translated into clinical AD research (Geldmacher et al., 2011; Sato et al., 2011); however, the associated phase III trials have been terminated due to poor efficacy (Galimberti and Scarpini, 2017; Dong et al., 2019).

Microglial inflammasome activation acts as a potent contributor to AD pathology, with a focus on NLRP3. The NLRP3 inflammasome, a multiprotein complex, contains the NLRP3 protein, adapter protein ASC, and procaspase-1. Studies have reported that direct or indirect inhibition of the NLRP3 inflammasome attenuates the microglial inflammatory responses in AD (Thawkar and Kaur, 2019). The efficacy of several NLRP3 inflammasome-targeting molecular inhibitors, including MCC950 (Dempsey et al., 2017; Lučiūnaitė et al., 2020) and JC-124 (Yin et al., 2018), has been confirmed by preclinical studies. Moreover, the usage of clinical drugs targeting the NLRP3 inflammasome has gained momentum. Minocycline is an anti-inflammatory tetracycline that can cross the blood-brain barrier (BBB). An *in vivo* study reported that minocycline reduces Aβ accumulation and attenuates microglial activation, possibly because of the inhibition of the NLRP3 inflammasome (Garcez et al., 2019). Clinical trials have also explored the neuroprotective effects of minocycline in AD treatment. However, in a previous study, targeting the inflammatory response by minocycline failed to delay the progression of cognitive impairment in patients with AD (Howard et al., 2020). Edaravone functions as a free radical scavenger and is widely used to treat cerebral infarction. Recent compelling evidence has highlighted the importance of edaravone’s anti-inflammatory effects on Aβ-stimulated microglial activation *via* inhibition of NLRP3 inflammasome activation (Wang et al., 2017). The neuroprotective role of edaravone has been confirmed by a series of *in vivo* and *in vitro* studies (Jiao et al., 2015; Yang et al., 2015; Wang et al., 2017; Parikh et al., 2018). In view of its efficient blood-brain barrier permeability and multiple action targets, edaravone seems to be a promising therapeutic agent.

Purinergic P2X receptor 7 (P2X7R), a member of the purinergic receptor, has promoted in-depth investigation into NLRP3 inflammasome activation. P2X7R is considered a potent activator of the NLRP3 inflammasome; both can be expressed by the microglia, and act synergistically to facilitate the release of pro-inflammatory mediators. Therefore, antagonizing P2X7R may mitigate the microglial inflammatory events in AD (Chiozzi et al., 2019; Thawkar and Kaur, 2019). In the brains of AD patients, P2X7R expression was colocalized with Aβ plaque-associated microglia, and the expression of microglial P2X7R was found to be increased in patients compared with controls (McLarnon et al., 2006). Consistently, these findings were confirmed in *in vitro* microglia cultures (McLarnon et al., 2006) and in AD transgenic mice (Lee et al., 2011). Furthermore, a specific inhibitor of P2X7R, oxidized ATP, was found to counteract microglial responses induced by co-stimulation with Aβ_1–42_ and selective P2X7R agonists (McLarnon et al., 2006). In an AD rodent model, another P2X7R inhibitor, brilliant blue G, was found to reduce microgliosis and to antagonize the inflammatory responses elicited by a P2X7R agonist (Ryu and McLarnon, 2008). Nimodipine, a dihydropyridine calcium channel antagonist, has also been shown to confer neuroprotective effects by reducing the levels of activated NF-κB and inhibiting the release of mature IL-1β in Aβ-stimulated microglia (whose potential target is P2X7R), which plays a permissive role in this process (Chiozzi et al., 2019). However, although P2X7R antagonists have sparked interest in the field of cellular and animal research, to date, no AD treatment pharmacotherapies have been incorporated into clinical trials.

As mentioned above, microglia-associated inflammatory events play pathological roles in AD progression, and a series of proteins and pathways are involved in this complex process. To date, many studies have investigated the molecular mechanisms underlying microglial inflammation in the context of AD. However, although these studies have yielded beneficial and effective results in cellular and transgenic animal models of AD, clinical trials targeting microglial inflammatory responses have reported limited success. A potential explanation is that microglia-associated inflammation is a multifactorial and complex process that is tightly interconnected, but the majority of studies tend to target an individual molecule or pathway to explore its potential therapeutic capacity. However, current cellular and transgenic murine models do not mimic the diverse aspects of AD, which explains why promising strategies in cellular and animal models have failed to exhibit efficacy in clinical trials.

## Conclusion

Although decades of investments and intense research have been conducted to elucidate the complete mechanism of AD neurodegeneration, there are still no effective therapies in terms of disease attenuation or prevention. The microglia are multifunctional immune cells in the brain that play a vital role in neurological disorders, especially in AD. The prolonged activation of microglia diminishes their phagocytic capabilities, releases a series of pro-inflammatory mediators, and aggravates Aβ and tau pathology in AD progression. The microglia-mediated pathological cascade is a highly complex process involving many proteins and pathways. Based on a thorough overview of the microglial pathology in AD, we learned that the potential therapeutic targets may focus on microglial modification, immunoreceptors, and inflammatory responses. Notably, the functional status of the microglia at specific stages of AD and the precise timing of microglial modifications are important factors that need to be discussed and evaluated. Further studies are also needed to explore the causal link between microglial pathology and AD in a more in-depth manner before the findings in mice can be translated into clinical treatment, as this may present promising avenues for therapeutic AD strategies.

## Author Contributions

GZ participated in drafting the manuscript. ZW, HH, and MZ reviewed the literature and revised the manuscript. LS designed and supervised the study. All authors contributed to the article and approved the submitted version.

## Conflict of Interest

The authors declare that the research was conducted in the absence of any commercial or financial relationships that could be construed as a potential conflict of interest.

## Publisher’s Note

All claims expressed in this article are solely those of the authors and do not necessarily represent those of their affiliated organizations, or those of the publisher, the editors and the reviewers. Any product that may be evaluated in this article, or claim that may be made by its manufacturer, is not guaranteed or endorsed by the publisher.
